# Suboptimal birth spacing practice and its predictors among reproductive-age women in Sub-Saharan African countries: a multilevel mixed-effects modeling with robust Poisson regression

**DOI:** 10.1186/s12978-023-01678-w

**Published:** 2023-09-04

**Authors:** Kusse Urmale Mare, Kebede Gemeda Sabo, Ahmed Adem Mohammed, Simeon Meskele Leyto, Getahun Fentaw Mulaw, Tsion Mulat Tebeje, Setognal Birara Aychiluhm, Oumer Abdulkadir Ebrahim, Abel Gebre Wuneh, Beminate Lemma Seifu

**Affiliations:** 1 Department of Nursing, College of Medicine and Health Sciences, Samara University, Samara, Ethiopia; 2 Department of Anatomy, College of Medicine and Health Sciences, Arba Minch University, Arba Minch, Ethiopia; 3 School of Public Health, College of Medicine and Health Sciences, Woldia University, Woldia, Ethiopia; 4 School of Public Health, College of Health Sciences and Medicine, Dilla University, Dilla, Ethiopia; 5 Department of Public Health, College of Medicine and Health Sciences, Samara University, Samara, Ethiopia

**Keywords:** Multilevel modeling, Reproductive-age women, Predictors, Robust Poisson regression, Suboptimal birth spacing, Sub-Saharan Africa

## Abstract

**Background:**

Despite the availability of exempted family planning services, a significant proportion of women in African countries continue to experience inadequately spaced pregnancies. To the authors’ knowledge, evidence of suboptimal birth intervals at the SSA level is lacking and previous studies have been limited to specific geographic area. Therefore, this analysis was aimed to estimate the pooled prevalence of suboptimal birth spacing and its predictors among childbearing women in SSA.

**Methods:**

Pooled DHS data from 35 SSA countries were used and a weighted sample of 221,098 reproductive-age women was considered in the analysis. The survey across all countries employed a cross-sectional study design and collected data on basic sociodemographic characteristics and different health indicators. Forest plot was used to present the overall and country-level prevalence of suboptimal birth spacing. Multilevel mixed-effects models with robust Poisson regression were fitted to identify the predictors of suboptimal birth spacing. Akaike’s and Bayesian information criteria and deviance were used to compare the models. In a multivariable regression model, a p-value less than 0.05 and an adjusted prevalence ratio with the corresponding 95% CI were used to assess the statistical significance of the explanatory variables.

**Results:**

The pooled prevalence of suboptimal birth spacing among women in SSA was 43.91% (43.71%-44.11%), with South Africa having the lowest prevalence (23.25%) and Chad having the highest (59.28%). It was also found that 14 of the 35 countries had a prevalence above the average for SSA. Rural residence [APR (95% CI) = 1.10 (1.12–1.15)], non-exposure to media [APR (95% CI) = 1.08 (1.07–1.11)], younger maternal age [APR (95% CI) = 2.05 (2.01–2.09)], non-use of contraception [APR (95% CI) = 1.18 (1.16–1.20)], unmet need for family planning [APR (95% CI) = 1.04 (1.03–1.06)], higher birth order [APR (95% CI) = 1.31 (1.28–1.34)], and desire to have at least six children [APR (95% CI) = 1.14 (1.13–1.16)] were the predictors of suboptimal birth spacing practice.

**Conclusion:**

More than four out of ten reproductive-age women in SSA countries gave birth to a subsequent child earlier than the recommended birth spacing, with considerable variations across the countries. Thus, interventions designed at enhancing optimal birth spacing should pay particular attention to young and socioeconomically disadvantaged women and those residing in rural regions. Strengthening community health programs and improving accessibility and availabilities of fertility control methods that ultimately impacts optimal reproductive behaviors is crucial to address contraceptive utilization and unmet need.

## Background

World Health Organization (WHO) defines a suboptimal birth interval as a duration of less than 33 months between two consecutive live births [1]. Proper timing and spacing of pregnancies is associated with a 25% reduction in mortality risk in children under-five years of age, that corresponds to an annual reduction of 1,836,000 deaths [2]. Conversely, a shorter interval between births has been linked with poor pregnancy and child health outcomes such as abortion and stillbirth, early neonatal and childhood deaths [2–9], preterm births, low birth weight [2, 5, 6, 8, 9], abnormal fetal position and presentation, low APGAR score, and respiratory distress syndrome [6, 8]. Furthermore, studies have shown that children born after a shorter preceding birth intervals are more likely to suffer from malnutrition (stunting, underweight, and anemia) [2, 6, 10].

In addition to adverse neonatal outcomes, closely spaced pregnancies have shown to have a significant effect on maternal health, particularly during pregnancy and childbirth. It has been revealed that women with shorter birth intervals are at a higher risk of preeclampsia [4, 6], anemia [11], hypertensive disorder [4, 8], premature rupture of membranes [4, 6], obstructed and prolonged labor [6, 8], hemorrhage, infection, and hospitalization [8].

Previous studies in different settings have reported a varying level of suboptimal birth spacing practice among reproductive-age women. Studies based on single-country data revealed that 23%, 26%, 47%, 49%, and 50% of reproductive-age women in Pakistan [12], Bangladesh [13], Ethiopia [14], Ghana [15], and rural India [16] respectively had experienced shorter birth interval. Furthermore, a secondary analysis of demographic and health survey (DHS) data showed that the prevalence of suboptimal birth spacing was 59% in ten high-fertility African countries [17] and 56% in thirteen Sub-Saharan African (SSA) countries [18].

Globally, the implementation of family planning program has witnessed a promising improvement in maternal and child survival in the multidimensional aspects, mainly by avoiding the risk of unintended and closely spaced pregnancies and its associated complication [19, 20]. In addition, the integration of family planning program with other maternal and child health services, provision of this service free of charge and post-partum family planning counseling, and the expansion of service delivery through community-based health programs were the other key initiatives being undertaken to enable women to use this service for achieving optimal pregnancy timing and spacing [21, 22].

However, despite the availability of exempted family planning services, a significant proportion of women in African countries continue to experience inadequately timed and spaced pregnancies [17, 18, 23] that puts them at a greater risk of morbidities and mortality related with pregnancy and childbirth. Therefore, information on the magnitude of suboptimal birth spacing and contextual factors influencing this maternal fertility behavior in these settings is important for redesigning the existing interventions and policy revision. To the authors’ knowledge, evidence of suboptimal birth intervals at the SSA level is lacking and previous studies have been limited to specific country or geographic area [14, 15, 24–26] and others have included only few African countries [17, 18, 23]. Thus, this analysis aimed to estimate the pooled prevalence of suboptimal birth spacing and its predictors among childbearing women in SSA using the most recent DHS data from 35 countries.

## Methods

### Study design, data source, and participants

DHS data of 35 sub-Saharan African countries were used in the present analysis. Countries were selected based on the survey year, availability of a standardized and unrestricted dataset, and presence of observations on the outcome variable in the datasets. For the current analysis, we included the countries that have their recent DHS conducted between 2010 and 2021. The survey across all countries employed a cross-sectional study design and collected data on basic sociodemographic characteristics and different health indicators.

All surveys used a multistage stratified cluster sampling technique to select the study participants. First, each country was divided into clusters, and clusters were randomly selected based on the probability proportional to their contribution to the overall country’s population. In the second stage, using the housing census as a sampling frame, a representative number of households was selected from each cluster. Survey data were collected using a standardized tool and face-to-face interviews. In the DHS, data on birth interval was collected by asking women about the interval between their last two successive live births. Thus, we have considered women of reproductive age who have had at least two births (second and higher order births) and those who had data on birth interval. On the contrary, women who were nulliparous, primiparous, had an abortion in between two live births, or had missing data for the birth interval variable were excluded from the study. For the current analysis, we used the women’s dataset (IR dataset), and a weighted sample of 221,098 reproductive-age women who had at least two successive live births was included in the final analysis. Details about DHS methodology can be accessed at (https://dhsprogram.com/Methodology/index.cfm).

### Variables and measurements

#### Data processing and statistical analysis

All data management procedures and analyses were performed using Stata version 17. Before analysis, the availability of the outcome variable in the DHS dataset of each country was confirmed and all variables considered in the study were checked for missing values. Then, the datasets of 35 SSA countries were appended and weighted to restore the representativeness of the sample and obtain reliable estimates and standard errors.

A multilevel mixed-effects Poisson regression model with robust error variance was fitted to identify the predictors of suboptimal birth spacing practice among reproductive-age women. We applied Poisson regression with robust error variance since the odds ratio estimated using a common binary outcome from cross-sectional data may significantly overestimate the strength of association [27, 28]. In addition, to account for the dependency of data due to the nested nature of DHS (i.e. women were nested within the households, and households were nested within the clusters), a multilevel mixed-effects logistic regression modeling was applied. Bivariable multilevel robust Poisson regression analysis was done and all variables with a p-value of less than 0.25 in this analysis were considered for multivariable multilevel robust Poisson regression model [29, 30].

In our analysis, four hierarchal models were fitted to select the model that best fits the data: a model with outcome variable only to assess the random variability in the intercept (model I), a model with individual-level explanatory variables (model II), a model with community-level explanatory variables (model III), and a model with both individual and community-level predictors (model IV). Akaike’s information criteria (AIC), Bayesian information criteria (BIC), Log-likelihood (LL), and deviance (i.e. -2*LL) values were used for model comparison. Model IV was selected as the best-fitted model since it had the lowest values on all three comparison parameters (AIC, BIC, and deviance). Random variability in suboptimal birth spacing practice among reproductive-age women across clusters was examined with random effect parameters like intra-class correlation coefficient (ICC), proportion change in variance (PCV), and median odds ratio (MOR). Collinearity diagnostic was assessed using variance inflation factor (VIF) and the VIF values for the variables included in the final regression analysis were less than five, suggesting that there was no significant multi-collinearity. In the final multivariable analysis, a p-value less than 0.05 and an adjusted prevalence ratio with the corresponding 95% confidence interval was used to identify the predictors of suboptimal birth spacing (Table 1).

**Table 1:** Study variables and their measurements

Variables	Measurements
Outcome variable	
Suboptimal birth spacing	In the survey dataset, this variable was recorded as the duration of the interval between the preceding and the most recent birth (in the number of months). For the analysis purpose, the variable was dichotomized based on the WHO recommendation using 33 months as a cut-off point. Thus, women with an interval of less than 33 months were considered to have “suboptimal birth spacing practice” (coded as “1”) and otherwise considered to have “optimal birth spacing practice” (coded as “0”) [1]
Independent variables	
Residence	Urban and Rural
Perception of distance to health facility	Not a big problem and Big problem
Current age	15–24, 25–34, and 35–49 year
Age at marriage	< 18 year and ≥ 18 year
Nature of marriage	Monogamous and Polygamous
Women’s education	No formal education, Primary education and Higher education
Husband’s/partner’s education	No formal education, Primary education and Higher education
Women’s working status	Unemployed and Employed
Sex of household head	Male and Female
Household wealth index	Poor, Middle, and Rich
Media exposure	Media exposure was created using three variables (television, radio, and newspapers) that have three response options (i.e. not at all, less than once a week, and at least once a week). Thus, women who reported watching television or listening to the radio, or reading the newspaper less than once a week and at least once a week were considered as having media exposure and otherwise labeled as not having exposure to mass media
Couple’s fertility preference	Women who reported that their husbands preferred to have the same number of children were regarded as having “concordant fertility preference”, while those whose partners desired to have less or more children than their desire were considered as having “discordant fertility preference”
Decision on health care utilization	This variable was generated by using the variable “who usually decides on women’s health care” that have four responses (respondent alone, respondent and partner, partner alone, and someone else). Thus, women were considered to have been “participation” if they reported that the decision was made by themselves or jointly with their partner and otherwise regarded as “not having participation”
Contraceptive use,	Yes and No
Unmet need for family planning	Yes and No
Birth order	≤ 3 and ≥ 4
Ideal number of children	≤ 5 and ≥ 6
History of pregnancy loss	Yes and No

#### Ethical considerations

We used publicly accessible DHS dataset and the survey procedures were approved by the ICF Institutional Review Board (IRB) and the host country IRB during the initial data collection. We have received permission to access the data from ICF International via online request.

## Results

### Participant’s characteristics

Of 221,098 reproductive-age women included in the analysis, 149,109 (67%) resided in rural dwellings, 86,073 (40%) perceived distance to the nearest health facility as a big problem, and 96,921 (44%) were between the ages of 25 and 34 years. Nearly half of the participants (49%) were married before the age of 18 and 53,361 (24%) were in a polygamous union. About two-thirds of women (65%) had exposure to mass media, 92,820 (43%) had no formal schooling, and 188,561 (85%) lived in male-headed households. Approximately half (51%) of women had ever used contraceptives and less than one-fourth (23%) of them had unmet family planning needs.

Our analysis also revealed that the proportion of suboptimal birth spacing was 46%, 47%, and 62% among women who resided in rural settings, those who did not participate in the decision to use healthcare services, and aged 15–24 years, respectively. Furthermore, suboptimal birth interval was relatively higher among women with no formal education (48%) compared to those who attended higher education (39%) (Table 2).

**Table 2:** Sociodemographic and obstetric characteristics of childbearing women in 35 SSA countries, 2010–2021

Characteristics	Weighted frequency	Weighted percentage	Proportion of suboptimal birth spacing (95% CI)
Residence			
Urban	71,989	32.56	39.76 (39.39, 40.12)
Rural	149,109	67.44	46.37 (46.12, 46.62)
Distance to a health facility			
Not a big problem	128,326	59.85	42.45 (42.18, 42.72)
Big problem	86,073	40.15	46.35 (46.02, 46.68)
Decision on healthcare utilization			
Has participation	127,054	57.49	41.86 (41.59, 42.13)
No participation	93,960	42.51	47.66 (47.34, 47.98)
Age at marriage			
≥ 18 year	112,755	51.00	42.42 (42.13, 42.71)
< 18 year	108,343	49.00	46.26 (45.97, 46.56)
Nature of marriage			
Monogamy	167,664	75.86	43.92 (43.68, 44.16)
Polygamy	53,361	24.14	45.48 (45.07, 45.89)
Current age			
15–24	28,636	12.95	62.13 (61.57, 62.69)
25–34	96,921	43.84	45.22 (44.91, 45.54)
35–49	95,541	43.21	38.07 (37.77, 38.38)
Women’s education			
No formal education	92,820	42.98	47.68 (47.36, 47.99)
Primary education	73,505	33.25	43.56 (43.20, 43.92)
Higher education	54,773	24.77	39.32 (38.90, 39.73)
Working status			
Unemployed	72,166	32.66	46.88 (46.52, 47.24)
Employed	148,777	67.34	
Husband education			
No formal education	82,996	37.55	47.57 (47.24, 47.91)
Primary education	64,429	29.16	44.47 (44.08, 44.85)
Higher education	73,528	33.28	40.35 (39.99, 40.71)
Media exposure			
Exposed	144,775	65.56	42.00 (41.75, 42.26)
Not exposed	76,069	34.44	48.40 (48.05, 48.75)
Household health			
Male	188,561	85.28	44.77 (44.55, 44.99)
Female	32,536	14.72	41.76 (41.23, 42.23)
Household wealth			
Rich	85,886	38.85	39.10 (38.77, 39.44)
Middle	44,793	20.26	44.49 (44.03, 44.96)
Poor	90,419	40.90	48.54 (48.23, 48.86)
Ever used contraceptives			
Yes	113,870	51.50	38.86 (38.57, 39.14)
No	107,228	48.50	49.83 (49.54, 50.13)
Unmet need for contraception			
No	169,492	76.67	43.40 (43.17, 43.64)
Yes	51,578	23.33	47.27 (46.84, 47.69)
Birth order			
≤ 3	91,556	41.41	42.39 (42.06, 42.71)
≥ 4	129,542	58.59	45.62 (45.36, 45.89)
Ideal number of children			
≤ 5	118,969	53.81	39.77 (39.49, 40.05)
≥ 6	102,129	46.19	49.29 (48.99, 49.60)
Couples fertility preferences			
Concordant	76,641	35.36	42.23 (41.89, 42.58)
Discordant	150,115	64.64	45.58 (45.32, 45.84)
History of pregnancy loss			
No	182,546	82.57	42.26 (45.03, 45.49)
Yes	38,526	17.43	39.76 (39.27, 40.25)

### Pooled prevalence of suboptimal birth spacing

**Fig. 1:**
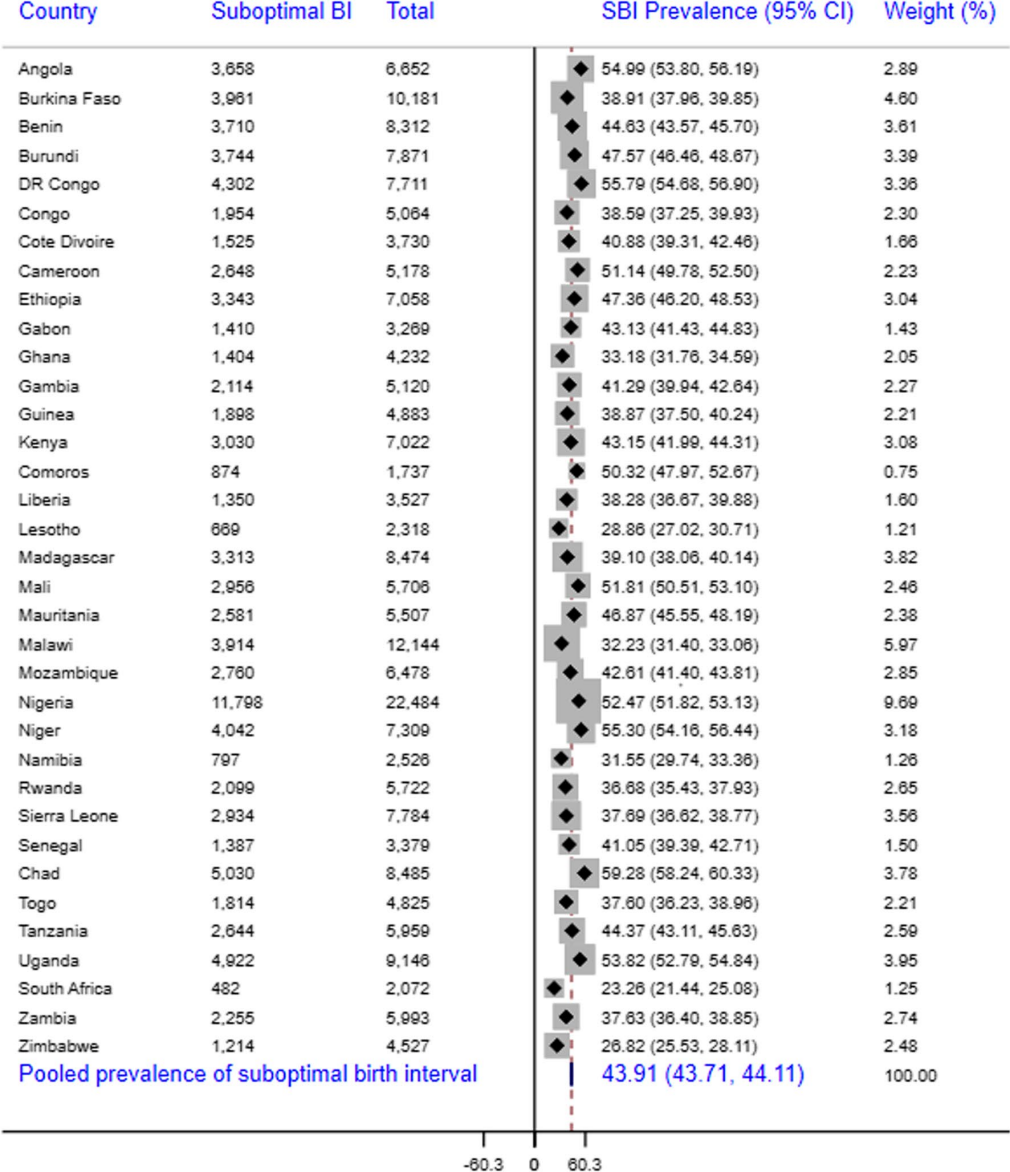
Country-level and pooled prevalence of suboptimal birth spacing among reproductive-age women in 35 SSA countries, 2010–2021

**Fig. 2:**
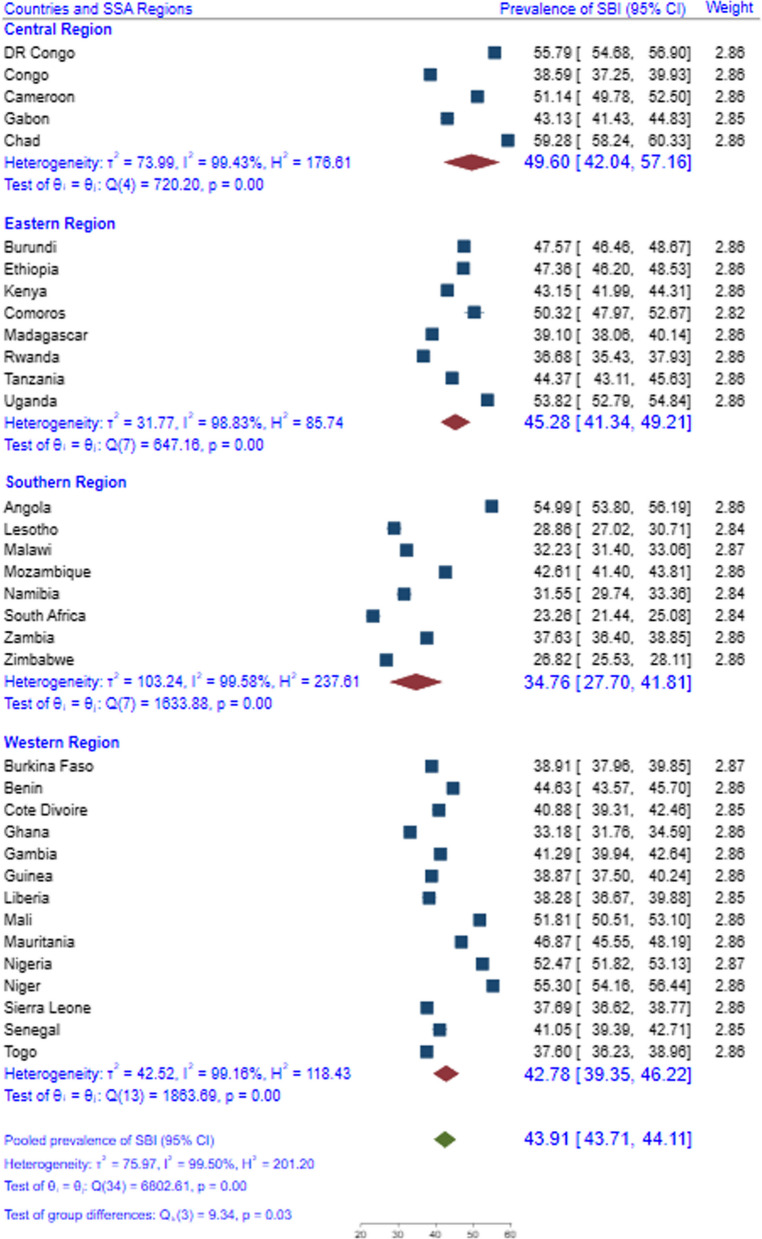
Subgroup analysis of suboptimal birth spacing among reproductive-age women across SSA regions, 2010–2021

The pooled prevalence of suboptimal birth spacing among childbearing women in SSA was 43.91% (95% CI = 43.71%, 44.11%), with South Africa having the lowest prevalence (23.26%) and Chad having the highest (59.28%). In addition, it was revealed that 14 of 35 countries included in the analysis had a prevalence greater than the average for SSA (43.91%) and six of these countries were from the Western African region (Fig. 1). This study also found that the Southern Africa region had the lowest magnitude of shorter birth spacing (34.76%), whereas Central Africa had the highest magnitude (49.60%), followed by Eastern (45.28%) and Western (42.78%) African regions (Fig. 2).

### Random-effect analysis result

In the null model (Model I), the ICC value indicated that about 14% of variation in suboptimal birth spacing practice was explained by differences across the clusters, while the remaining 86% was attributed to individual-level differences. In the final model, the values of explained variance also showed that about 35% of the total variation in shorter birth interval was attributed to the combined effect of individual and community-level factors. In addition, the presence of heterogeneity in the level of suboptimal birth interval across the clusters was indicated by the MOR of 1.90 and 1.58 in the null and full models, respectively. This shows that women living in the cluster with a higher prevalence of suboptimal birth interval had a 90% higher likelihood of experiencing closely spaced births compared to those in the clusters with a lower prevalence of shorter birth spacing. Model IV had the lowest AIC, BIC, and deviance values and was hence selected as the best-fitted model (Table 3).

**Table 3:** Random intercept models (measures of variation) at cluster or community level for suboptimal birth spacing practice among reproductive-age women in 35 SSA countries, 2010–2021

Measure of variation	Model 1	Model 2	Model 3	Model 4
Cluster-level variance (%)	57%	34%	41%	37%
Intra-class correlation (%)	14.10%	10.12%	11.08%	10.10%
Explained variance (%)	Reference	40.35%	28.07%	35.09%
Median odds ratio	1.90	1.51	1.66	1.58
Model statistics summary				
Akaike’s information criteria	354,246	347,143	342,077	336,040
Bayesian information criteria	354,267	347,277	342,129	336,204
Log-likelihood	− 177,121	− 173,559	− 171,034	− 168,004
Deviance	354,242	347,118	342,068	336,008

### Predictors of suboptimal birth spacing (Fixed-effect analysis result)

After adjusting for the effect of confounders, the result of multivariable multilevel robust Poisson regression analysis revealed that place of residence, age, husband education, media exposure, household wealth, contraceptive use, unmet family planning need, birth order, and an ideal number of children were the significant predictors of suboptimal birth spacing. We found that women who lived in rural areas [APR (95% CI) = 1.10 (1.12–1.15)] and those who did not have media exposure [APR (95% CI) = 1.08 (1.07–1.11)] had a higher prevalence of suboptimal birth interval compared to their counterparts. Women aged 15–24 years [APR (95% CI) = 2.05 (2.01–2.09)] and 25–34 years [APR (95% CI) = 1.31 (1.29–1.33)], and those with no formal education [APR (95%) = 1.04 (1.02–1.06)] and primary level education [APR (95% CI = 1.02 (1.01–1.04)] had a greater risk of having shorter birth interval than their reference groups. Compared to women in the richer households, those from families with middle [APR (95% CI) = 1.03 (1.01–1.05)] and poor [APR (95%) = 1.06 (1.05–1.08)] wealth indexes had a higher prevalence of suboptimal birth interval.

Furthermore, our result showed that women who had never used contraception [APR (95% CI) = 1.18 (1.16–1.20)] and unmet family planning need [APR (95% CI) = 1.04 (1.03–1.06)] had an increased risk of suboptimal birth interval compared to those who ever used fertility control methods and those who did not have an unmet contraceptive need, respectively. Additionally, the likelihood of experiencing suboptimal birth interval was significantly greater for women with higher birth order [APR (95% CI) = 1.31 (1.28–1.34)] and those who desire to have six children or more [APR (95% CI) = 1.14 (1.13–1.16)] (Table 4).

**Table 4:** Predictors of suboptimal birth spacing among childbearing women in 35 SSA countries, 2010–2021

Covariates	Birth Spacing		UPR (95% CI)	APR (95% CI)
	Optimal	Suboptimal		
Residence				
Urban	43,847 (35.4)	28,142 (28.9)	1.00	1.00
Rural	79,989 (64.6)	69,120 (71.1)	1.19 (1.16, 1.20)	1.10 (1.12, 1.15)*
Distance to a health facility				
Not a big problem	73,971 (61.3)	54,355 (57.9)	1.00	1.00
Big problem	46,627 (38.7)	39,446 (42.1)	1.08 (1.06, 1.09)	1.00 (0.98, 1.05)
Decision on healthcare utilization				
Has participation	74,644 (60.3)	52,410 (53.9)	1.00	1.00
No participation	49,145 (39.7)	44,815 (46.1)	1.16 (1.14, 1.17)	1.02 (0.99, 1.04)
Current age				
35–49	59,480 (48.0)	36,061 (37.1)	1.00	1.00
25–34	53,470 (43.2)	43,451 (44.7)	1.19 (1.17, 1.20)	1.31(1.29, 1.33)*
15–24	10,886 (8.8)	17,750 (18.2)	1.64 (1.62, 1.17)	2.05 (2.01, 2.09)*
Husband education				
Higher education	44,293 (35.8)	29,236 (30.1)	1.00	1.00
Primary education	35,833 (28.9)	28,596 (29.4)	1.12 (1.09, 1.13)	1.02 (1.01, 1.04)*
No formal education	43,607 (35.2)	39,359 (40.5)	1.19 (1.17, 1.21)	1.04 (1.02, 1.06)*
Media exposure				
Exposed	84,205 (68.1)	60,570 (62.4)	1.00	1.00
Not exposed	39,504 (31.9)	36,565 (37.6)	1.14 (1.13, 1.16)	1.08 (1.07, 1.11)*
Household wealth				
Rich	52,529 (42.4)	33,357 (34.3)	1.00	1.00
Middle	24,832 (20.1)	19,962 (20.5)	1.15 (1.13, 1.17)	1.03 (1.01, 1.05)*
Poor	46,475 (37.5)	43,943 (45.2)	1.25 (1.23, 1.27)	1.06 (1.05, 1.08)*
Ever used contraceptives				
Yes	69,869 (56.4)	44,001 (45.2)	1.00	1.00
No	53,967 (43.6)	53,261 (54.8)	1.28 (1.27, 1.30)	1.18 (1.16, 1.20)*
Unmet family planning need				
No	96,509 (77.9)	72,982 (75.1)	1.00	1,00
Yes	27,310 (22.1)	24,268 (24.9)	1.09 (1.08, 1.11)	1.04 (1.03, 1.06)*
Birth order				
≤ 3	53,168 (42.9)	38,388 (39.5)	1.00	1.00
≥ 4	70,668 (57.1)	58,874 (60.5)	1.08 (1.07, 1.10)	1.31 (1.28, 1.34)*
Ideal number of children				
≤ 5	71,975 (58.1)	46,994 (48.3)	1.00	1.00
≥ 6	51,861 (41.9)	50,268 (51.7)	1.25 (1.23, 1.26)	1.14 (1.13, 1.16)*

*UPR* unadjusted prevalence ratio; *APR* adjusted prevalence ratio; *statistically significant variables at *p*-value less than 0.05

## Discussion

In the present analysis, nationally representative demographic and health survey data from 35 Sub-Saharan African countries were used to estimate the pooled prevalence and predictors of suboptimal birth spacing among childbearing women. Our analysis revealed that the overall prevalence of suboptimal birth spacing among women in SSA was 43.9% (43.7%-44.1%), with considerable within-country variations from 23.3% in South Africa to 59.3% in Chad. The level of suboptimal birth spacing practice observed in this study is higher than the prevalence reported from the studies conducted in Pakistan (23%) [12], Bangladesh (26%) [13], and rural India (50%) [16] but lower than the finding of systematic review in Ethiopia (47%) [14], the studies in ten high fertility African countries (59%) [17], and thirteen SSA countries (56%) [18]. Variations in the level of suboptimal birth spacing across the studies might be attributed to differences in the population characteristics, religious and sociocultural contexts, access to reproductive health services like contraception and other fertility-related services, and differences in healthcare infrastructure across the settings.

The result of a multilevel robust Poisson regression analysis showed that suboptimal birth spacing practice was significantly influenced by different socio-demographic and reproductive characteristics. For instance, compared to women who resided in urban settings, rural women had a higher prevalence of shorter birth spacing, which is consistent with the findings of the previous studies [15–17, 24, 26]. A higher risk of shorter birth intervals among rural women might be linked to lower contraceptive knowledge and limited access to contraceptive services and health information in rural settings [31, 32]. In addition, the finding might also be explained by urban–rural differences in the socio-cultural contexts and geographic access to health facilities that deter maternal utilization of modern fertility control methods, particularly in rural areas [33].

The current study also showed that shorter birth intervals were more likely to occur among younger mothers. Women between the ages of 25 and 34 years had a 31% higher prevalence of suboptimal birth interval than those aged 35 to 49 years, and the risk was increased by about two-fold for younger women (15–24 years). This finding is in agreement with the result of the previous studies that reported older maternal age as a protective factor for short birth spacing [12, 13, 15, 16, 23, 34]. This might be attributed to the fact that younger women have inadequate reproductive knowledge [35] and limited participation in the decision regarding contraceptive use and therefore more likely to experience closely spaced births than older women [36, 37]. Low socioeconomic status of younger women that hinder them from accessing the means and information to achieve optimal child spacing could also be the most possible justification for this finding [38].

Contraceptive utilization was also identified as a significant predictor of birth spacing among reproductive-age women. Compared to women who had a history of using contraceptives, women who had never used contraceptives were 18% more likely to experience a second birth after an interval of less than 33 months. The results of the earlier studies are in accordance with this finding [12, 14, 16, 17, 25, 34], where non-use of contraceptives was reported as an enabling factor for experiencing suboptimal birth spacing. Our result supports already established fact about the impact of family planning programs in reducing high-risk fertility indices like short birth intervals [39].

Consistent with the previous studies [15, 34, 40], birth order was also found as an important factor in predicting the occurrence of closely spaced births among reproductive-age women. Women with a parity of four or more had a greater prevalence of suboptimally timed births than those with lower birth orders. The possible justification for this finding is that multiparous women are less likely to use fertility control methods and thus tend to have shorter birth spacing than their counterparts [41, 42].

Additionally, our analysis showed that compared to reproductive-age women who desired to have fewer children, women who wanted to have more than five children had a 14% increased risk of experiencing shorter birth intervals. Similarly, previous studies also reported a higher likelihood of shorter birth spacing with an increasing maternal desire to have more children [17, 24]. This might be because women who want more children are likely to have repeated and closely spaced pregnancies to achieve their fertility preferences. Furthermore, the direct statistical relationship between maternal fertility desire and lower contraceptive utilization could be a plausible justification for this finding [43].

The result of this study also revealed that women who did not have exposure to mass media were more likely to have a suboptimal birth spacing practice than those who had exposure to such information sources. This result is consistent with a previous study in Ethiopia that reported a 35% added odds of shorter birth interval among women unexposed to media [24]. The most possible justification for this finding is that exposed women have better knowledge and awareness of maternal healthcare services and are thus more likely to practice healthy reproductive behavior than their reference group [44, 45].

## Strengths and limitations

The use of a larger sample size, nationally representative data from 35 countries, and advanced statistical methods are the main strengths of this study. However, it is impossible to explain the causal relationship between the independent and dependent variables due to the cross-sectional nature of the survey’s design. There might also be a recall bias since women were asked about the events that took place five years or more preceding the survey.

## Conclusion

This study showed that more than four out of ten reproductive-age women in SSA countries gave birth to the subsequent child earlier than the recommended waiting time, with considerable variations in the level of this practice across the countries. The result also revealed that rural residency, younger maternal age, low husband education, non-exposure to mass media, poor household wealth, non-use of contraceptives, unmet family planning need, higher birth order, and ideal number of children were the significant predictors of suboptimal birth spacing. Therefore, interventions designed at enhancing optimal birth spacing should pay particular attention to young and socioeconomically disadvantaged women and those residing in rural regions. Establishing regular reproductive health education programs through mass media and outreach activities is also important to increase awareness of the ideal timing of pregnancies. Additionally, strengthening community health programs and improving accessibility and availabilities of fertility control methods that ultimately impacts optimal reproductive behaviors is crucial to address contraceptive utilization and unmet need.

## Data Availability

The raw dataset used and analyzed in this study can be accessed from the DHS website (http://www.measuredhs.com).

## Abbreviations

AIC: Akaike’s Information Criteria
APR: Adjusted Prevalence Ratio
BIC: Bayesian Information Criteria
CI: Confidence Interval
DHS: Demographic and Health Survey
ICC: Intra Class Correlation Coefficient
IRB: Institutional Review Board
LL: Log-Likelihood
MOR: Median Odds Ratio
PCV: Proportional Change in Variance
SSA: Sub-Saharan Africa
UPR: Unadjusted Prevalence Ratio
VIF: Variance Inflation Factor
WHO: World Health Organization
